# Prediction of paravalvular leakage after transcatheter aortic valve implantation

**DOI:** 10.1007/s10554-015-0703-1

**Published:** 2015-07-18

**Authors:** Luigi F. M. Di Martino, Wim B. Vletter, Ben Ren, Carl Schultz, Nicolas M. Van Mieghem, Osama I. I. Soliman, Matteo Di Biase, Peter P. de Jaegere, Marcel L. Geleijnse

**Affiliations:** From the Department of Cardiology, Ospedali Riuniti, Università degli studi di Foggia, Foggia, Italy; From the Department of Cardiology, Erasmus University Medical Center, Thoraxcenter, Ba304, ’s-Gravendijkwal 230, 3015 CE Rotterdam, The Netherlands; From the Cardialysis Cardiovascular Core Laboratory, Rotterdam, The Netherlands

**Keywords:** Aortic valve, Transcatheter, Computed tomography, Echocardiography, Paravalvular leakage, TAVI

## Abstract

Significant paravalvular leakage (PVL) after transcatheter aortic valve implantation (TAVI) is related to patient mortality. Predicting the development of PVL has focused on computed tomography (CT) derived variables but literature targeting CoreValve devices is limited, controversial, and did not make use of standardized echocardiographic methods. The study included 164 consecutive patients with severe aortic stenosis that underwent TAVI with a Medtronic CoreValve system©, with available pre-TAVI CT and pre-discharge transthoracic echocardiography. The predictive value for significant PVL of the CT-derived Agatston score, aortic annulus size and eccentricity, and “cover index” was assessed, according to both echocardiographic Valve Academic Research Consortium (VARC) criteria and angiographic Sellers criteria. Univariate predictors for more than mild PVL were the maximal diameter of the aortic annulus size (for both angiographic and echocardiographic assessment of PVL), cover index (for echocardiographic assessment of PVL only), and Agatston score (for both angiographic and echocardiographic assessment of PVL). The aortic annulus eccentricity index was not predicting PVL. At multivariate analysis, Agatston score was the only independent predictor for both angiographic and echocardiographic assessment of PVL. Agatston score is the only independent predictor of PVL regardless of the used imaging technique for the definition of PVL.

## Introduction

Paravalvular aortic leakage (PVL) after transcatheter aortic valve implantation (TAVI) is a complication with potentially severe consequences [1–6]. The main focus in predicting the development of PVL has been on computed tomography (CT) derived variables such as calcium quantification with the Agatston score, aortic annulus size and eccentricity, and indexes relating the annulus dimensions to prosthesis size like the “cover index” [3]. However, prediction of PVL may be different for the various percutaneous valves, since the CoreValve Revalving System© is self-expandable while the Edwards SAPIEN™ prosthesis is a balloon-expandable one. In particular, in a self-expandable prosthesis, calcified native valves may pose resistance to deployment, resulting in an ellipsoid-shaped stent and a higher incidence of PVL. Indeed, Agatston score was predictive for PVL in all published CoreValve specific studies [7–9]. However, literature focusing on CoreValve devices and other CT-derived predictors is limited and reported results are discrepant. The value of aortic annulus eccentricity was investigated in only one small study [10] and aortic annulus size or the “cover index” was only analyzed in two relatively small CoreValve studies with conflicting results [10, 11]. One of the reasons for these discrepancies may be the different methods of defining the PVL end-point: by angiography [10] versus pre-discharge transthoracic echocardiography [11]. Also, echocardiographic assessment of PVL was not performed according to the recently updated Valve Academic Research Consortium (VARC-2) criteria [12]. The current study sought to assess, in the thus far largest published single-center consecutive CoreValve series, the univariate and multivariate predictive value of CT-derived Agatston score, aortic annulus size and eccentricity, and “cover index” for significant PVL. PVL was defined according to both echocardiographic VARC-2 criteria (also for the first time defined as a continuous variable rather than a categorical variable) and angiographic Sellers criteria.

## Methods

### Patients

The study included 164 consecutive patients with severe aortic stenosis that underwent TAVI with a Medtronic CoreValve system© from June 2006 to November 2012, with available pre-operative Agatston score and pre-discharge transthoracic echocardiography. The details of the TAVI implantation procedure are described in full detail elsewhere [13–15]. The first five patients underwent TAVI with the second-generation Medtronic CoreValve delivery system, which is implanted using a 21Fr catheter inserted into the common femoral (n = 4) or the subclavian (n = 1) artery using surgical exposure without the use of an arterial sheath. All other patients underwent TAVI with the third-generation delivery system, using an 18Fr arterial sheath inserted into the femoral artery using an echocardiographic-guided Seldinger technique and closure with a 10Fr Prostar7 (Prostar XL, Abbott Vascular, IL); except for four who underwent the subclavian approach. All patients underwent general anesthesia, and valve implantation was done using cine and fluoroscopic guidance. The institutional review board approved the study.

### CT study

A pre-operative CT scan was performed in all patients using dual source CT (Somatom Definition, Siemens Medical Solutions, Forchheim, Germany). A non-contrast calcification score acquisition was performed before contrast MSCT. The pitch was adjusted to fit the heart rate, and the volume of iodinated contrast material was adapted to the expected scan time: 50–60 ml of VisipaqueVR 320 mg l/ml, (GE Health Care, Eindhoven, The Netherlands) were injected in an antecubital vein at a flow rate of 5.0 ml/s followed by a second contrast bolus of 30–40 at 3.0 ml/s. The scan ranged from the top of the aortic arch to the diaphragm. 3D reconstructions in end systole were obtained using a single-segmental algorithm with slice thickness 1.5 mm and increment 0.4 mm. The radiation doses ranged from 8 to 20 mSv depending on body habitus and table speed. The aortic annulus was defined as a virtual ring with three anchor points at the bases of the three aortic leaflets [16]; the minimum and maximum diameters and area of the annulus were measured in a viewing plane axial to the aortic root to match that definition [17]. Propriety software was developed to allow measurement of aortic annulus size on a contrast MSCT (3mensio Medical Imaging, Bilthoven, the Netherlands). A scan without contrast enhancement was available in 98 of 110 patients because it was initially not performed in patients with previous CABG or coronary stents. The non-contrast MSCT acquisition was performed in a prospectively ECG-triggered, sequential (step-and-shoot) mode with a reference tube current of 80 mAs, a tube voltage of 120 kV and slice thickness of 3 mm in the early or mid diastolic heart phase depending on the heart rate; the latter data sets were used to derive the Agatstone score [18]. For analysis on a dedicated cardiovascular CT workstation (MMWP, Siemens AG, Forchheim, Germany) the aortic root was defined as the stretching from the caudal aspect of the aortic annulus to the origin of the left main stem as seen on axial images [19]. The threshold for the detection of calcium was set at 130 HU. In cases where aortic root cal-cification was confluent with calcium in adjacent structures (mitral annulus, ascending aorta, coronary arteries) only the stack of images that contained the aortic root were selected.

### Angiographic evaluation of paravalvular leakage

Ten minutes after the deployment of the prosthetic valve, angiography of the aortic root was performed to assess the severity of aortic regurgitation according to Sellers criteria [20]. During evaluation of the aortography images the cases were labeled according to the following criteria: (0) no regurgitation; (1) only trace of contrast could be seen in the left ventricle, and it is cleared in each systole; (2) contrast filling the entire LV in diastole with less density compared with opacification of the ascending aorta; (3) contrast filling the entire LV in diastole equal in density to the contrast opacification of the ascending aorta; and (4) contrast filling of the entire LV in diastole on the first beat with greater density compared with the contrast opacification of the ascending aorta. Two observers independently scored the images. In case of discrepancy the images were re-evaluated and consensus was reached by a third observer.

### Echocardiographic study

All patients were evaluated after TAVI by pre-discharge transthoracic echocardiography using an iE33 ultrasound system (Philips Medical System, Best, the Netherlands) equipped with a S5-1 transducer. The extent of PVL was assessed according to the main VARC criterion, that is the circumferential extent of PVL in a parasternal short-axis view [12], as seen in Fig. 3. The VARC scores were provided as continuous values as well as categorical values: (0) no regurgitation; (1) mild PVL was defined as <10 % circumferential extent; (2) moderate PVL was defined as >10 % but <30 % of PVL and (3) severe PVL was defined as >30 % according to the updated VARC guideline [12]. Significant PVL was defined as a VARC-2 score more than mild.

### Predictors of PVL

Maximal aortic annulus diameter [3] was obtained from the CT scans using three-dimensional reconstruction performed with the Siemens Circulation© software, in a plane aligned to cut the lower part of all three the aortic cusps, as described earlier by us [11]. The Cover Index was defined as $100 \times \, (nominal\,prosthesis \, diameter - CT \, mean \, annulus \, diameter)/nominal \, prosthesis \, diameter$ [21, 22], and the Eccentricity Index was calculated as $100 \, \times (1 - (aortic \, annulus \, minimum \, diameter/maximum \, diameter))$ [10]. The annulus measurements for the latter indices were derived from the same CT plane described before, and the mean diameter was calculated as an average between the maximum and minimum one. The prosthesis nominal diameters used were provided by the manufacturer. The amount of calcification [7–9] was assessed as the Agatston score by the same CT analysis software in the non-contrast scans, with the interest zone confined to the segment of the aorta ranging from the anterior mitral leaflet to the origin of the left coronary artery.

### Statistical analysis

All data gathered were analyzed using SPSS (IBM, version 20). Continuous variables were checked for normal distribution via the Kolmogorov–Smirnov test and were expressed as mean (±standard deviation), median (25–75 % percentile), or number (percentage) as appropriate. Inter and intraobserver variability was expressed as correlation as well as weighted kappa for categories. The predictors were plotted against both the continuous values of VARC-2 score and the Sellers degree using Spearman correlation; *p* values and Spearman’s coefficient (ρ) are provided. Because of a lack of homogeneity between the VARC-2 score, divided in four categories, and the Sellers’ one, that recognizes five categories, the latter was compared with the continuous values of circumferential extent of the PVL. Each predictor was also tested against significant (more than mild) paravalvular regurgitation by Mann–Whitney test. Multivariate analysis was carried on by linear regression with a stepwise backward method.

## Results

### Clinical and demographic characteristics of the population

Of the 164 patients, 87 were male (54 %), and the median age was 81 (78–85) years. Pre-operative aortic mean pressure gradient was 43 ± 15 mm Hg, and aortic valve area was 0.7 ± 0.2 cm^2^. (Table 1) The Logistic Euroscore median was 13 (10–21). A 26 mm device was implanted in 42 (26 %) patients, a 29 mm in 113 (69 %) and a 31 mm in 9 (5 %) cases.

**Table 1:** Clinical characteristics of the study population

Feature	Value
Age (years), median (IQR)	81 (78–85)
Male, n (%)	87 (54)
New York Heart Association class ≥III, n (%)	132 (81)
Previous cerebrovascular event, n (%)	39 (24)
Previous myocardial infarction, n (%)	39 (24)
Previous coronary artery bypass graft surgery, n (%)	49 (30)
Previous percutaneous coronary intervention, n (%)	44 (27)
Diabetes mellitus, n (%)	43 (27)
Hypertension, n (%)	98 (60)
Peripheral vascular disease, n (%)	19 (12)
Chronic obstructive pulmonary disease, n (%)	43 (27)
Laboratory results	
Creatinine (umol/l), median (IQR)	93 (74–118)
Haemoglobin (g/dl), median (IQR)	7.7 (7.1–8.4)
Logistic euroscore, median (IQR)	13 (10–21)
Mean aortic pressure gradient (mmHg), mean ± SD	43 ± 15
Aortic valve area (cm^2^), mean ± SD	0.7 ± 0.2

### Inter and intraobserver variability in the angiographic and echocardiographic evaluation

Inter and intraobserver variability in the echocardiographic evaluation was assessed in a subset of 50 random patients. The same clips were evaluated by two experienced echocardiographers to provide an esteem of the interobserver variability (r = 0,92, *p* < 0,05 for continuous values) and were then re-evaluated in random order after some months by one of them to assess intraobserver variability (r = 0,95, *p* < 0,05). Categorical values were also not statistically different in interobserver variability (weighted κ = 0.86; see Table 2).

**Table 2:** Category correlation for interobserver variability in VARC score in a subset of 50 random patients: weighted κ = 0.86 (0.66–0.93)

Observer 1					
Grade	0	+1	+2	+3	Total
Observer 2					
0	19	1	0	0	20
+1	0	12	2	0	14
+2	0	2	11	1	14
+3	0	0	1	1	2
Total	19	15	14	2	50

0 = none, 1 = mild, 2 = moderate, 3 = severe

Correlation in the Sellers’ score series between the first and the second observer was acceptable (r = 0,87, *p* < 0.05); cathegorical values are provided for the same subset of patients selected for the previous analysis (weighted κ = 0.90, see Table 3). To provide a better evaluation, however, a consensus was obtained from a third observer in case of disagreement and the resulting scores were used for further statistical analysis.

**Table 3:** Category correlation for interobserver variability in Sellers’ score in a subset of 50 random patients: weighted κ = 0.90 (0.74–0.99)

Observer 1						
Grade	0	+1	+2	+3	+4	Total
Observer 2						
0	5	0	0	0	0	5
+1	0	11	1	0	0	14
+2	0	2	26	0	0	14
+3	0	0	1	4	0	5
+4	0	0	0	0	0	0
Total	5	13	28	4	0	50

### Correlation between echocardiographic and angiographic evaluation of PVL

**Fig. 1:**
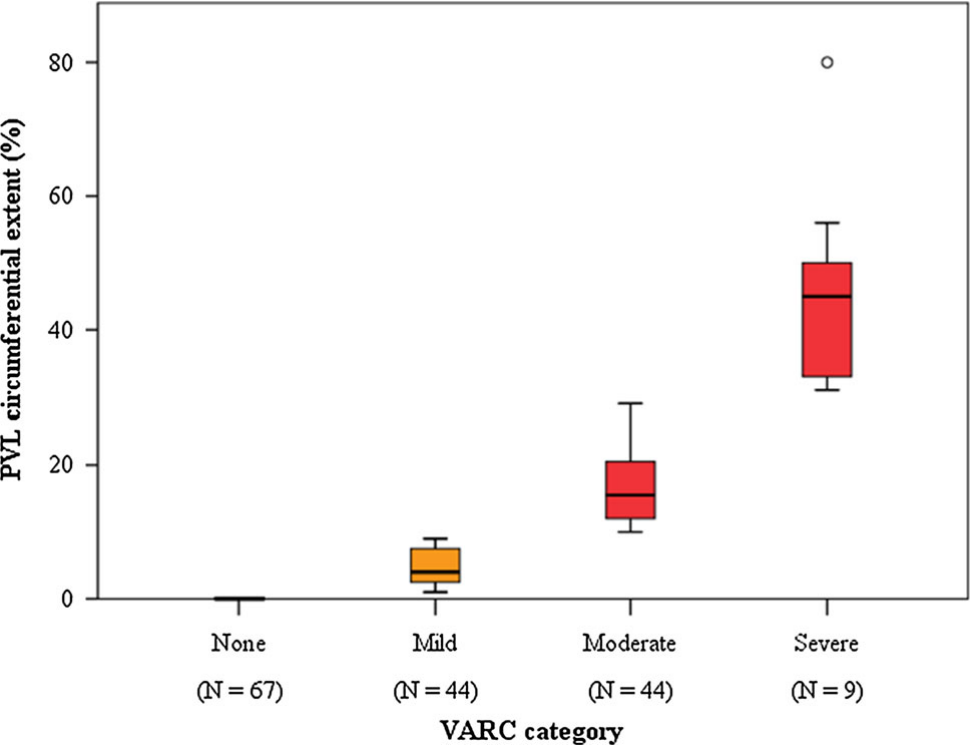
Distribution of VARC-2 scores according to the VARC-2 categories

**Fig. 2:**
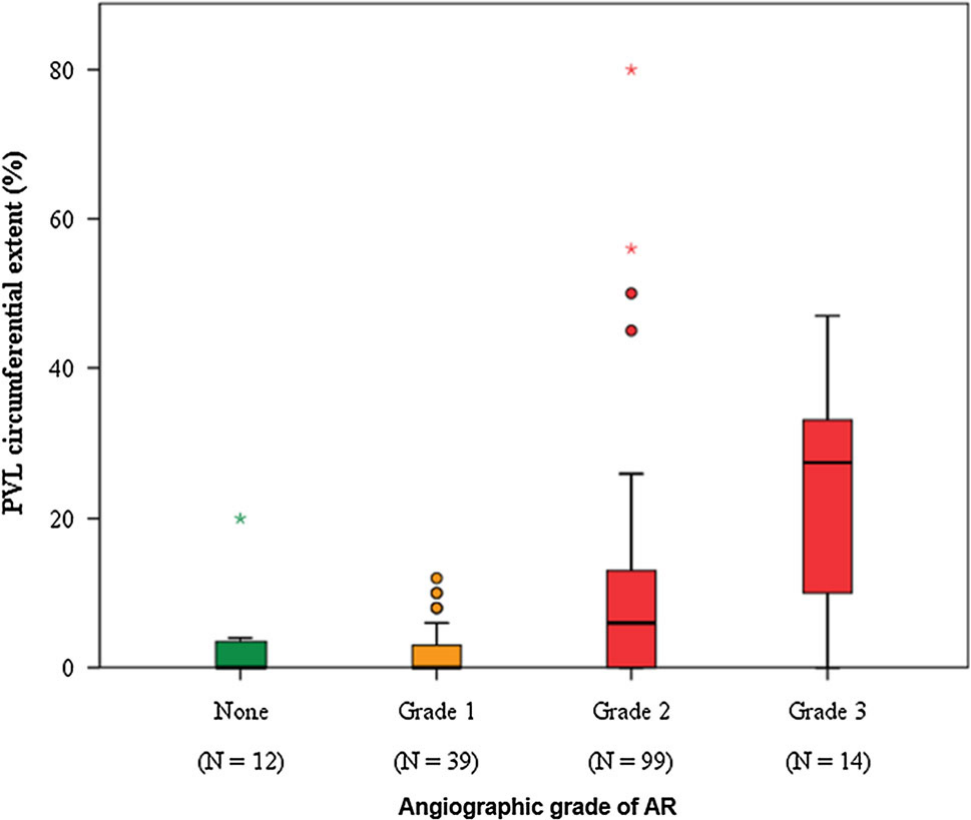
Correlation between angiographic Sellers grading and continuous value of VARC-2 scores for the assessment of paravalvular leakage

At echocardiography PVL was not observed in 67 patients (41 %); it was mild in 44 (27 %), moderate in 44 (27 %), and severe in 9 (5 %). More than mild PVL was thus detected in 53 cases (32 %). The median values (25-75 percentiles) in the categories were 4 % (3–8 %) for mild, 16 % (12–21 %) for moderate and 45 % (33–50 %) for severe PVL (Fig. 1). Following Sellers criteria, the patients were classified as aortic regurgitation grade 0 in 12 (7 %) cases, grade 1 in 39 (24 %), grade 2 in 99 (60 %), and grade 3 in 14 (9 %). No patient had grade 4 aortic regurgitation. There was a significant correlation between the Sellers and VARC score (ρ = 0.481, *p* < 0.001) (Fig. 2).

### Correlation between aortic annulus and cover index

There was a significant inverse correlation (ρ = −0.734, *p* < 0.001) between the maximum aortic annulus diameter and the cover index.

### Predictors of PVL as assessed by angiography (Sellers)

As seen in Tables 4 and 5, significant predictors for more than mild PVL assessed by angiography were the maximal diameter of the aortic annulus size (27.9 ± 2.6 vs. 26.7 ± 2.3 mm, *p* = 0.006; correlation 0.178, *p* = 0.005), cover index (12.6 [9.6–15.1] vs. 14.4 [11.8–18.2], *p* = 0.007; correlation −0.143, *p* = 0.019]), and Agatston score (3,346 [2,363–4886] vs. 2123 [1477–2777] Hounsfield units, *p* < 0.001; correlation 0.395, *p* < 0.001). The eccentricity index was not a predictor for PVL.

**Table 4:** Prediction of aortic paravalvular leakage as assessed by echocardiography (VARC-2) and angiography (Sellers)

Predictor	Echocardiographic VARC-2 score			Angiographic Sellers score		
	None or mild n = 111	More than mild n = 53	*p* value	None or mild n = 51	More than mild n = 113	*p* value
Maximal annulus diameter, mm	27.2 ± 2.5	28.1 ± 2.4	0.039	26.7 ± 2.3	27.9 ± 2.6	0.006
Cover index (%)	14.0 (10.3–17.3)	12.6 (8.6–16.4)	0.204	14.4 (11.8–18.2)	12.6 (9.6–15.1)	0.007
Eccentricity index (%)	20.4 ± 6.7	20.9 ± 6.4	0.644	20.5 ± 6.4	20.6 ± 6.7	0.939
Agatston score	2596 (1782–4034)	3952 (2528–5071)	0.001	2123 (1477–2777)	3346 (2363–4886)	<0.001

**Table 5:** Correlations between predictors for aortic paravalvular leakage and actual paravalvular leakage as assessed by echocardiography (VARC-2) and angiography (Sellers)

Predictor	VARC-2 score		Angiography score	
	ρ value	*p* value	ρ value	*p* value
Maximal annulus diameter	0.210	0.003	0.178	0.005
Cover index	–0.134	0.043	–0.143	0.019
Eccentricity index	0.030	0.350	0.036	0.303
Agatston score	0.305	<0.001	0.395	<0.001

### Predictors of PVL as assessed by echocardiography (VARC-2)

As seen in Tables 4 and 5, significant predictors for more than mild PVL assessed by echocardiography were the maximal diameter of the aortic annulus size (28.1 ± 2.4 vs. 27.2 ± 2.5 mm, *p* = 0.039; correlation 0.210, *p* = 0.003), and Agatston score (3952 [2528–5071] vs. 2596 [1782–4034] Hounsfield units, *p* = 0.001; correlation 0.305, *p* < 0.001). Although not a significant predictor, a weak but significant correlation existed between the cover index and PVL (correlation −0.134, *p* = 0.043). The eccentricity index was not a predictor for PVL.

### Multivariate analysis

**Fig. 3:**
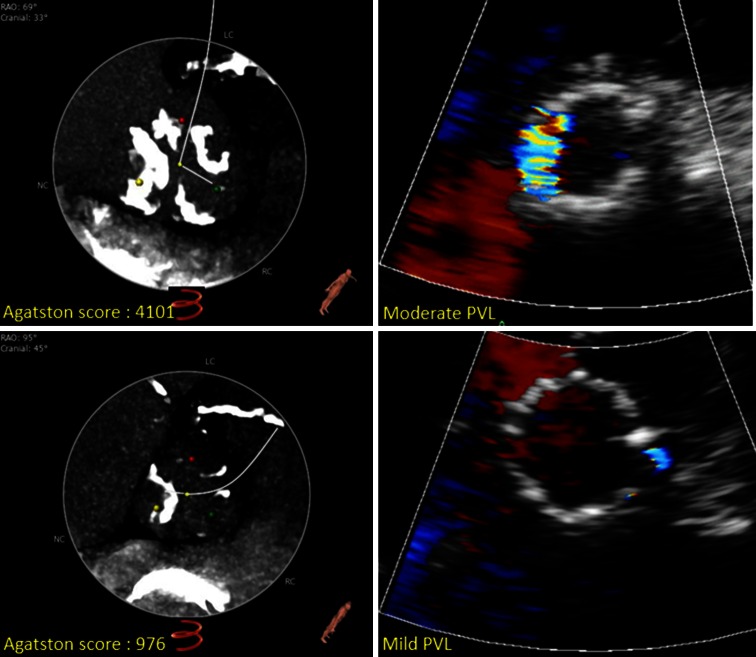
Examples from two patients with low and high Agatston score, respectively and corresponding *colour* Doppler short-axis views of paravalvular leak on 2D echocardiography

At multivariate analysis, the Agatston score was the only independent predictor for PVL, regardless of the method of PVL assessment: *p* = 0.001, β = 0.265 for the echocardiographic VARC score and *p* = 0.004, β = 0.272 for the angiographic Sellers score (Fig. 3).

## Discussion

The main findings of this study are: (1) maximal aortic annulus diameter and Agatston score predicted more than mild PVL and correlated to PVL extent regardless of the used imaging technique to define PVL, (2) cover index predicted angiographic PVL but did not predict echocardiographic PVL, although a weak but significant correlation existed, (3) aortic annulus eccentricity did not predict PVL, and (4) Agatston score was for both PVL imaging techniques the only independent predictor for PVL.

The literature about prediction of PVL after implantation of a CoreValve prosthesis is limited because of the relatively small number of available CoreValve specific reports [7, 8, 10, 11, 23, 24], including small number of patients, conflicting results, assessment of PVL by different techniques (angiography [10] versus pre-discharge transthoracic echocardiography [11]), and the use of echocardiographic methods not recommended by the VARC [3, 25]. The present study is the largest so far published single-center consecutive CoreValve series describing prediction of PVL as defined by both angiographic and VARC echocardiography criteria with well-known parameters as the Agatston score, aortic annulus size and eccentricity, and the cover index. Also, correlations between predictors and outcome (PVL) parameters were for the first time based on both continuous variables and categorical variables.

Aortic annulus calcification measured by Agatston score was predictive for PVL in all published CoreValve specific studies [7, 8, 11] and in our study it was the only independent predictor of PVL, regardless of its definition by angiography or echocardiography. The relationship between aortic annulus calcification and the incidence of PVL is based on a suboptimal adherence of the prosthesis to the aortic root walls because of the calcified native leaflets that, even if crushed by balloon inflation before, during, or after the procedure, cannot be totally removed. The powerful Agatston score was recently reported to even predict cardiovascular events [7].

In addition, lower values of the cover index were correlated with the severity of PVL, confirming that a prosthesis that is too small related to the native annulus can induce PVL. This has already led to a tendency to oversize the prosthesis [26]. The relationship between the larger size of the aortic annulus and the incidence of PVL is probably due to a greater probability of undersizing of the prosthetic valve; patients with a larger aortic annulus had indeed lower cover index values. It should also be noted that only at the final time frame of this study the largest (31 mm) CoreValve prosthesis size became available. Although not an independent predictor for PVL it should be recognised that the cover index is in fact the only one on which the interventional cardiologist can actually intervene, since all others are strictly related to the anatomy of the patient.

The aortic annulus is well known to be eccentric [27] but the eccentricity index did not predict PVL. Its value recently already met some criticism [28]. It is known that, according to CT scans before and after TAVI with balloon-expandable devices, the aortic annulus becomes more circular after the prosthesis placement, adapting to its shape [22, 29, 30]. Therefore, it may be expected that the annular shape may influence the grade of PVL only to a small extent. However, the CoreValve prosthesis is self-expandable and may adapt more to the annulus rather than vice versa. Our data show that also with the CoreValve prosthesis aortic annulus eccentricity does not influence the grade of PVL, although it cannot be excluded that balloon inflation performed before the implantation in patients with a significant calcium burden may have influenced this result.

### Limitations

The Sellers score includes total aortic regurgitation (transvalvular and paravalvular cannot be separately assessed) which will always to some extent limit the correlation to an echocardiographic PVL score. However, transvalvular aortic regurgitation was seen on echocardiography in a very small amount of patients and was usually trivial.

Both the Sellers score and the VARC score are highly subjective. In particular, the echocardiographic VARC one is not validated and harbours many difficulties such as underestimation due to poor acoustic windows or an incorrect (to high) level of image acquisition, or overestimation due to circumferentially flying jets and an incorrect (to low) level of image acquisition.

## Conclusion

The Agatston score is the only independent predictor of PVL regardless of the used imaging technique for the definition of PVL.
